# Assessment of Testifying Ability in Preschool Children: CAPALIST

**DOI:** 10.3389/fpsyg.2021.662630

**Published:** 2021-07-16

**Authors:** María José Contreras, Gerardo Prieto, Eva A. Silva, José L. González, Antonio L. Manzanero

**Affiliations:** ^1^Departamento de Psicología Básica I, Facultad de Psicología, Universidad Nacional de Educación a Distancia (UNED), Madrid, Spain; ^2^Departamento de Psicología Básica, Psicobiología y Metodología de las Ciencias del Comportamiento, Facultad de Psicología, Universidad de Salamanca, Salamanca, Spain; ^3^Guardia Civil, Ministerio del Interior, Gobierno de España, Madrid, Spain; ^4^Departamento de Psicología Experimental, Procesos Cognitivos y Logopedia, Facultad de Psicología, Universidad Complutense de Madrid, Madrid, Spain

**Keywords:** testifying ability, preschool children, CAPALIST, eyewitness, credibility assessment

## Abstract

**Purpose**: Interviews to obtain statements in judicial procedures need to be adapted to the witnesses’ abilities to testify. Moreover, knowing the cognitive abilities involved in testifying provides relevant criteria to assess statement credibility. As age or intelligence quotient is not enough to estimate these capabilities, an instrument to evaluate witnesses’ specific abilities to testify is needed. The present paper validates CAPALIST, a procedure that considers relevant capabilities when assessing the testimony given by children.

**Methods**: This study analyzed, by means of an invariant measurement approach (Rasch model), four scales included in CAPALIST: language, memory, contextual information, and social thinking. In addition, gender and age differences were analyzed in 83 children [45 males and 38 females; *M*_age_ = 4.3 years, *SD* = 0.74, range (3.06–5.11)] from three courses in early childhood education.

**Results**: The four scales do not severely violate the requirements of the model. The principal component analysis of the residuals indicates that the four scales are one dimensional and that the assumption of local independence was not violated. Differential item functioning of the scales associated with gender was not detected. A significant effect of the school year was obtained, with an increase in ability in successive courses. The percentage of children who presented severe misfit responses with the model was low. In addition, the number of items with a severe misfit was also low.

**Conclusion**: An acceptable performance of CAPALIST is demonstrated for most of the scales, although items with a severe misfit must be replaced, and more difficult items have to be included in some scales of the revised version of the instrument. CAPALIST is a promising procedure to assess the abilities of children to testify in order to adapt interviews and to evaluate their statements correctly.

## Introduction

The Convention on the Rights of the Child (UN, 1989) placed obligations on States to follow the principles of a child-friendly justice. The Optional Protocol on the sale of children, child prostitution, and child pornography (A/RES/54/263) established in Article 8 states that the State Parties shall adopt appropriate measures to protect the rights and interests of child victims from the practices prohibited under the present Protocol at all stages of the criminal justice process, in particular by recognizing the vulnerability of child victims and adapting procedures to recognize their special needs, including their special needs as witnesses. In order to comply with this mandate, it becomes essential to adapt the procedures used to take witness and victim statements to their abilities. Moreover, in the procedures used to analyze the statement credibility of children (for example, in cases of child sexual abuse), it is considered essential to know the cognitive abilities of witnesses and victims when assessing the credibility criteria (Volbert and Steller, 2014; Köhnken et al., 2015). The presence of certain details in the statements (contextual embedding, interaction languages, attribution of a perpetrator’s mental state, etc.) may depend on the cognitive development of a child (Manzanero et al., 2019). Lack of detail in a statement is wrongly considered to indicate low credibility when it could be due to the child’s skills to provide details. This may result in the rejection of testimonies of children with poor testifying skills rather than adapting the procedures to achieve higher-quality testimonies.

Age or intelligence quotient (IQ) is not enough to estimate these capabilities. Cognitive development varies among children of the same age. Also, different studies have shown a low relationship between IQ and the ability to provide a valid testimony (Kebbell and Hatton, 1999; Manzanero et al., 2012). However, no tools have been developed to assess the specific skills required to make a high-quality statement. In this way, the credibility assessment of testimonies provided by children and people with intellectual disabilities is carried out based on stereotypes, which usually leads to errors (Valenti-Hein and Schwartz, 1993; Bottoms et al., 2003; Henry et al., 2011; Manzanero et al., 2015).

For this reason, the CAPALIST (List of Capabilities Instrument, Silva et al., 2016, 2018) instrument was developed, as there was no procedure to carry out the assessment with a minimum of guarantees, which resulted in cases with this type of victims being frequently archived, with a stereotypical presumption of low credibility of testimony. The CAPALIST Protocol has entailed an advance in the proposal of police and judicial procedures that do not rule out the possibilities of obtaining truthful testimony from those who cannot be assumed to have a minimum level of these abilities. Previous work has described in detail the need for the assessment of abilities in minors such as language and memory (Silva et al., 2016), the validation of the protocol in minors (Silva et al., 2018), and a case study on the application of the protocol among people with intellectual disabilities (Contreras et al., 2015); thus, the need for an instrument such as the CAPALIST will not be reiterated here. However, an analysis of the instrument is necessary from the point of view of the functioning of the items proposed so far in order to refine and improve the instrument for its application in police and judicial spheres.

The objective of this work is to validate the CAPALIST protocol, using invariant measurement models, with the aim of discovering its strengths and weaknesses through a psychometric methodology in order to propose changes for the final version. Invariant measurement models, such as the Rasch model, constitute an operationalization of the axioms of additive conjoint measurement (Rasch, 1960; Tennant and Conaghan, 2007; Engelhard, 2013). If the data do not violate the requirements of the model, they make it possible to measure people and items on an interval scale to contrast the one-dimensionality of the scale that is necessary to justify the sum of the items and to detect the invariance of the items between subgroups of the sample. Current approaches in the construction of psychometric tests complement the traditional methods with the use of analysis with some of the Rasch-type models.

## Materials and Methods

### Participants

Eighty-three children from the three courses of preschool education participated in the study: 22 students from the first year (9 girls and 13 boys), 29 from the second year (15 girls and 14 boys), and 32 from the third year (14 girls and 18 boys). The mean age was 4.3 years [*SD* = 0.74; range (3.06–5.11)]. Written informed consent to participate in this study was provided by the legal guardians of the participants.

### Instruments

CAPALIST was used to evaluate testifying abilities. The instrument consists of different blocks of questions, organized into four large variables that are the object of analysis in the present study: language, memory, social thinking, and contextual information. In the complete instrument (see Appendix I), they are ordered as they are presented when applying the protocol. To administer the instrument, the use of the drawing (see Appendix II) from the *Short Procedure for the Assessment of the Abilities to Testify* is required (Manzanero and González, 2015; González and Manzanero, 2018).^1^

The items for each of the variables and the constructs they evaluate are detailed below:

1. *Language* (L1 to L22): Items that assess the interviewee’s ability to describe, referring to questions about:
  – Events in terms of knowing how to answer questions, such as who, where, what. In other words, questions about:
  – People: Is he/she able to differentiate between acquaintances and strangers?
  – Places: Is he/she able to point out where he/she is?
  – Objects: Is he/she able to identify certain animate or inanimate objects?
2. *Memory* (M1 to M15): These are items related to autobiographical and episodic memory, which assess a child’s ability to provide data related to his/her life, family, and the ability to remember more or less recent episodes.
3. *Social Thinking*: The following sets of items are included in this block:
  – Assertiveness (A1–A6): Misleading or suggestible items to assess the participant’s vulnerability to be induced toward a response by the evaluator. These controls that assess assertiveness are important to evaluate the resistance of the interviewee to a certain deceptive bias.
  – Subjective States (ES1 to ES8): Identification of states related to emotions, both the participant’s own emotions or other people’s emotions.
  – Moral Ability (CM1–CM6): These items evaluate sets of actions of people from the point of view of goodness or malice, related to a set of norms that regulate human behavior according to certain values.
4. *Contextual information*: This scale is divided into the following blocks of items:
  – Spatial orientation (OE1–OE8): Questions regarding events that have occurred in a certain space – where. Is he/she able to position himself/herself in the current space?
  – Temporal orientation (OT1–OT9): Questions regarding events in a certain time – when – and more specifically, to distinguish between:
    Present: Is he/she able to identify day/month/year of the time of the interview?
    Past: Is he/she able to indicate the day/month/year of the events reported or of another event in the recent past?
  – Numerical (N1–N13): Questions related to the participant’s ability to talk about quantities and numbers – how many. Is he/she able to differentiate between many and few?

The instrument was applied during an individual interview. The responses were scored with a polytomous Likert-type format with three categories: “1” if a child does not have the ability or present difficulty in answering the question, “2” if the answer corresponds to a basic ability, and “3” in those cases in which the answer entails mastery of the ability at the time of answering.

### Data Analyses

The rating scale model (RSM, Andrich, 1978), a Rasch-type model for polytomous items, was used initially to treat data obtained with Likert-type categories (Prieto and Delgado, 2007). The basic equation states that

$$\ln\;\left(P_{nik}/P_{ni\left(k-1\right)}\right)=B_{n}-D_{i}-F_{k}$$

where

*P_nik_* is the probability that the response of person *n* to the item *i* is scored with category *k*,

*P_ni(k−1)_* is the probability that the response of person *n* to the item *i* is scored with a category lower than *k* (*k*−*1*),

*B_n_* is the level of person *n* in the measured attribute,

*D_i_* is the level of item *i* in the measured attribute,

*F_k_* is the value of the variable in which the adjacent categories (*k* and *k*−*1*) have the same probability of being used. *F_k_* is known as a step or a threshold, where the number of steps is equal to the number of categories minus 1.

A central aspect of the rating scale model is its usefulness to empirically verify the functionality of Likert-type categories. The categories are considered adequate if they meet the criteria defined by Linacre (2002), among which the ordering of the thresholds across the adjacent categories (*F_k_*) stands out. According to Robinson et al. (2019), the threshold is the point at which a person has the same probability of being scored in adjacent response categories. When the use of a category is not the most probable in a certain range of the variable, the thresholds are out of order, and the probability curve of the category appears flatter than the rest. Excess of categories or a deficient definition of such categories can produce the disorder of the thresholds and, consequently, the inconsistency of the responses of the participants. The deficiency could be solvable by collapsing adjacent categories. As indicated in the results section, disordered thresholds appeared in three of the CAPALIST variables; thus, it was decided to use the Rasch (1960) dichotomous model after grouping categories 1 and 2 into one.

According to Pallant and Tennant (2007), the dichotomous model assumes that the probability that a given person correctly solves an item is a logistic function of the relative distance between the locations of the item (D) and the respondent (B) in the latent scale. The model is described in the formula: ln(*P_ni_*/1–*P_ni_*) = *B_n_*–*D_i_*, in which *ln* is the natural logarithm, *P* is the probability that a subject *n* hits item *i*, *B* is the person’s level in the latent variable, and *D* is the location or difficulty of the item in that dimension.

The model allows us to transform the ordinal values of the responses to a scale of intervals, called “*logit*,” in which the location parameters of the people (*B*) and the items (*D*) are located in the same dimension. Conventionally, 0 on the scale is placed in the average of the item parameters. For both, people and items, values greater than 0 indicate a higher level in the measured attribute (for example, memory) and, inversely, values less than 0 indicate a lower level. The closeness to zero of the mean of the people indicates the adaptation of the test to the level in the attribute of the sample.

The joint measurement in the same dimension of the people and the items makes it easier to analyze their interaction, determining the probability that a person masters an item based on the magnitude of the difference between his or her level in the measured variable and the location of the item in the variable. This property allows us to build norms referring to the latent variable, in addition to the traditional norms referring to the group.

The precision or reliability of the parameters of the items and of the people is estimated at the group level, using the statistics known as the item reliability index (IRI) and the person reliability index (PRI). Both statistics vary between 0 and 1. IRI indicates the precision with which the location of the items in the measured attribute, and the replicability of its parameters in other samples of people has been estimated. PRI has a meaning analogous to Cronbach’s alpha coefficient (proportion of the observed variance of the participants that is not associated with the variance of error) and indicates the precision with which the level of the participants is estimated in the measured attribute. The *separation index* (*G*) and the *strata index* (S) are other precision statistics at the group level that can be applied to people (*G_p_*; *S_p_*) and to items (*G_i_*; *S_i_*). *G*, which can range from 0 to +∞, is the quotient between the reproducible standard deviation from the model and the average of the standard errors of measurement. *S* is calculated from *G*: (4*G*+1)/3. It indicates the number of reliably different strata (of people or items) that can be identified in the data. That is, *S_i_* indicates the number of strata of the items that reliably present different difficulties, and *S_p_* is the number of strata of people that reliably present different levels in the measured attribute (Bond and Fox, 2015).

The requirements that the model imposes on the data are invariance of the parameters, one-dimensionality, and local independence.

The invariance of the parameters refers to the fact that the scale must work in the same way, regardless of the group being evaluated (Engelhard, 2013). That is, except for a linear transformation, the parameters of the people will be invariant to the sample of items used, and the parameters of the items will be invariant to the sample of people used for the calibration. The invariance of the parameters was called *specific objectivity* by Rasch (Prieto and Delgado, 2003).

A consequence of the assumption of invariance is the absence of differential item functioning (DIF), associated with groups of people formed by biological, social, or cultural variables that have the same level in the measured variable. For example, when measuring *contextual information*, males and females with the same level of spatial and temporal orientation should have the same probability of hitting an item. That is, the probability of a response must be conditioned only by the trait measured and not by other different characteristics associated with gender or culture (Pallant and Tennant, 2007). In sum, the presence of DIF represents a violation of the assumption of invariance and a threat to the validity of the measures.

Due to the small sample sizes in the present study, only the uniform DIF associated with gender has been analyzed. The uniform DIF occurs when one of the groups has a greater probability of obtaining hits across the measured variable (for example, males with low-, medium-, or high-memory levels are more likely to obtain a hit on an item than females in the same levels).

To detect the presence of uniform DIF, the difference between the difficulty parameters of each item between the focus group and the comparison group is calculated, calibrating the test for the entire sample. The DIF must be taken into consideration when the difference is greater than 0.64 logit and if it is statistically significant (Linacre, 2019). Welch’s t with Bonferroni’s correction is used as a contrast statistic: The difference is significant if the probability is less than 0.05/number of contrasts (Prieto and Nieto, 2014).

Residual means (differences between observed and expected values) are used to assess the fit of the data to the above-mentioned model requirements: outfit (the unweighted mean of the squares of the standardized residuals) and infit (the weighted mean with the variance of the squares of the standardized residuals). According to Linacre (2019), the *infit* and *outfit* values equivalent to the unit indicate a perfect fit; those higher than 2.0 indicate a severe misfit that invalidates the measure and the values that range between 1.5 and 2.0 represent a moderate misfit that does not have serious consequences for the validity of the measures.

Apart from the fit statistics, the principal component analysis of the residuals is usually used to test the one-dimensionality of the measures. The data are considered to be fundamentally one dimensional if the Rasch dimension explains more than 20% of the variance of the data (Reckase, 1979), and if, after controlling for this dimension, no significant patterns appear in the residuals. It is considered that there is no relevant secondary dimension when the eigenvalue of the first component of the residuals is less than 3 (Chou and Wang, 2010; Linacre, 2019).

Local independence is a requirement of the model; the violation of which can be assessed through dependence of the responses. Response dependency occurs when the items are linked in such a way that the response to one item determines the response to another item. Therefore, the probability of the response does not basically depend on the difference between the person and the item in the dimension extracted by the model. The local dependency of the items can be derived from the redundancy of the content that increases the homogeneity and spuriously inflates the reliability indicators such as internal consistency (for example, Cronbach’s alpha coefficient).

From the matrix of correlations between the residuals, it is possible to identify the item pairs with local dependence (LD). Yen (1984) proposed a LD statistic called *Q*_3_, which is the correlation between the residuals of item pairs. In the present study, similarly to Christensen et al. (2017) criterion, item pairs with a *Q*_3_ value greater than 0.20 were considered to show local dependence. If there is a violation of the local independence requirement, some of the dependent items may have to be removed.

The Winsteps program (version 4.4.1) was used to analyze the data (Linacre, 2019).

## Results

The data from the initial version were analyzed with the rating scale model. Table 1 shows the thresholds between the successive response categories in the four CAPALIST variables. It is noteworthy that the thresholds were out of order in the memory, social thinking, and contextual orientation variables. Likewise, the characteristic curves of the variable categories are shown as Supplementary Figures 1–4. Supplementary Figures 2–4 show that category 2 is not modal: It is not the most likely choice in any range of the measured variable. Therefore, the decision was made to add the two lower categories in the four variables, looking for uniformity of the scoring system: 1 = masters the ability (old category 3); 0 = does not adequately master the ability (old categories 1 and 2). The data obtained with this recoding were analyzed with the Rasch dichotomous model.

**Table 1:** Thresholds between successive response categories (F1-F2).

Threshold	Language	Memory	Social thinking	Context
F1	−0.64	0.61	0.89	0.38
F2	0.64	−0.61	−0.89	−0.38

### Analysis of the Language Variable With the Dichotomous Model

**Figure 1:**
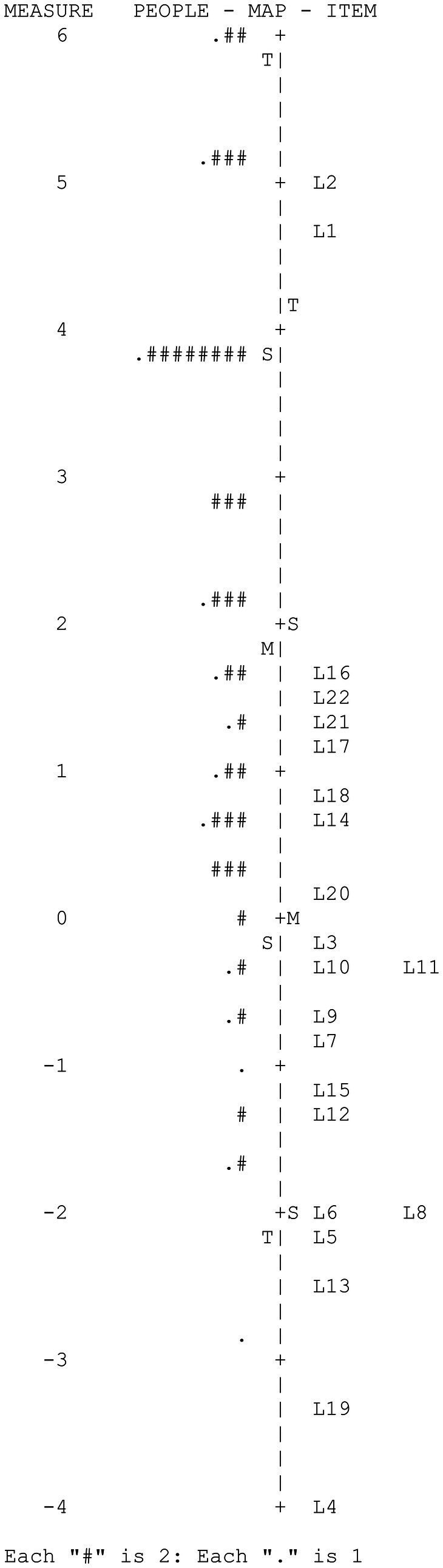
CAPALIST-language. Variable map.

Figure 1 shows the Wright map corresponding to the *language* variable. The map is a joint representation by means of a double vertical histogram of the people’s ability and item difficulty (Wilson, 2005). The map provides two basic results: (1) high variability of the participants and of the items in the measured variable and (2) the low adaptation of the difficulty of some items to the level of the examinees due to their extreme ease.

Table 2 shows the values of the items. Despite the fact that the people sample size was not large, the item difficulty parameters were estimated with high precision: The reliability index of the items was high (IRI = 0.94). The strata index of the items (S_i_ = 5.54) indicated that more than five ranges of items with different difficulties were reliably identifiable.

**Table 2:** CAPALIST-language. Item properties.

Item	X	D	SE	Infit	Outfit	RiX
1.L1. What is your teacher like? (Minimum 2 elements)	17	4.59	0.37	1.01	1.35	0.57
2.L2. Now, close your eyes and describe me (description of the interviewer Minimum 2 elements)	14	5.03	0.40	0.88	0.57	0.61
3.L3. Can you tell me what that girl is wearing? Start from the bottom. (If he/she does not know how, interviewer will point items out)	65	−0.18	0.33	0.96	1.35	0.52
4.L4. Tell me which ones are boys and which are girls in this picture (give them names)	83	−5.37	1.85	--	--	0.00
5.L5. Who is the tallest?	78	−2.21	0.51	1.00	0.75	0.31
6.L6. And the shortest?	77	−1.96	0.48	1.08	0.78	0.32
7.L7. Coloring the picture…. What is this color? And this one? (minimum 5 colors)	71	−0.92	0.37	1.07	0.83	0.44
8.L8. What can you see in the picture? Minimum 5 elements	77	−1.96	0.48	0.73	0.27	0.43
9.L9. And this boy, can you tell me what this boy is wearing? Start from the bottom. (If he/she does not know how, interviewer will point items out). Minimum X elements	69	−0.65	0.36	1.06	1.72	0.45
10.L10. Now I’m going to point out parts of the children’s bodies and you must tell me what they are called, ok?	67	−0.41	0.34	1.48	3.32	0.32
11.L11. Where are the children in the picture?	66	−0.29	0.34	1.15	3.56	0.45
12.L12. And in this picture, can you describe what the house is like?	74	−1.38	0.41	0.90	0.62	0.44
13.L13. What is that in the tree?	79	−2.50	0.56	1.24	0.89	0.22
14.L14. What things are there in your class?	57	0.63	0.31	0.78	0.58	0.67
15.L15. What things are there in your bedroom?	73	−1.21	0.40	0.96	0.50	0.45
16.L16. Describe the gym at your school	46	1.65	0.30	0.84	0.94	0.70
17.L17. What is the supermarket where you go shopping with mam and dad like?	51	1.19	0.30	1.06	0.92	0.63
18.L18. What is the park that you go to play like?	54	0.91	0.31	0.77	0.53	0.70
19.L19. What is in your backpack?	81	−3.33	0.76	1.24	1.63	0.11
20.L20. What is your jacket/coat/jumper like?	62	0.14	0.32	1.11	0.89	0.54
21.L21. What is the bathroom that is closest to your classroom like?	50	1.28	0.30	0.91	0.64	0.68
22.L22. Describe a kitchen	47	1.56	0.30	0.68	0.87	0.74
Mean	61.7	−0.24	0.46	0.99	1.12	0.47
SD	18.3	2.32	0.32	0.19	0.83	0.20
Max.	83	5.03	1.85	1.48	3.56	0.74
Min.	14	−5.37	0.30	0.68	0.27	0.00

The item difficulty in logit varied between 5.03 and −5.37 (*M* = −0.24; *SD* = 2.32). Item 4 was correctly solved by the entire sample, which is why its difficulty parameter was extremely low and its discrimination was null (RiX = 0.00). This discriminative inefficiency is why the program automatically excluded the item from the variable and placed the origin of the scale at the mean of the remaining 21 items. In that case, the items varied in difficulty between 5.03 and −3.33. Two items (L10 and L11) presented an outfit value greater than 2, indicating a severe misfit with the model.

The principal component analysis of the residuals indicated that the subscale can be considered one dimensional, since the measurements accounted for a sufficient percentage of the total variance (53.7%) and the eigenvalue of the first component of the residuals were less than 3 (2.15). On the other hand, it was observed that only three of the 231 values of *Q*_3_ were higher than 0.20 (1.3%), which indicates that local dependence does not seriously affect the items. The maximum value of *Q*_3_ was 0.59 (correlation between the residuals of items L3 and L9). Furthermore, no gender-related DIF was detected in any of the items.

Table 3 shows the descriptive statistics of the evaluated scores: The scores ranged between 22 and 4 hits and 6.69 and −2.80 logits. The standard deviations of the hits and the logits were high and indicated that the evaluated scores varied greatly in the measured variable. It is noteworthy that the mean in logits of the participants (2.12) was much higher than the mean difficulty of the items (−0.24), indicating that the test was easy for the children evaluated. In fact, it was observed that six extremely easy items were correctly solved by more than 90% of the evaluated individuals (L4, L5, L6, L8, L13, and L19). Table 3 also shows the means in the logit scale of the groups formed according to sex and grade. The average levels of females and males did not differ significantly, *t*(79) = 1.65, *p* = 0.10. However, performance grew significantly with the school grade, *F* (2, 80) = 27.15, *p* < 0.001. The mean for second-year students was higher than that for first-year students by 1.40 logits, this difference being significant, *t* (42) = 2.68, *p* < 0.01, and of medium effect size (*d* = 0.78); the mean for third-year students was higher than that for second-year students by 2.11 logits, with this difference being significant, *t* (58) = 4.91, *p* < 0.001 and with a large effect size (*d* = 1.28).

**Table 3:** Language. Descriptive statistics of the participants’ scores.

Group	Mean X	SD X	Max. X	Min. X	Alpha	Mean L	SD L	Max. L	Min. L	PSR
Total	16.4	4.3	22	4	0.87	2.12	2.23	6.69	−2.80	0.79
Male	15.6	4.7	22	4	0.88	1.76	2.25	6.69	−2.80	0.80
Female	17.3	3.7	22	7	0.86	2.56	2.13	6.69	−1.60	0.77
1st Year	12.4	4.3	22	4	0.84	0.30	1.91	6.69	−2.80	0.85
2nd Year	16.0	3.6	21	7	0.81	1.71	1.71	5.12	−1.60	0.76
3rd Year	19.6	1.8	22	15	0.56	3.82	1.59	6.69	0.95	0.33

X, hits; L, logit; Alpha, Cronbach’s alpha coefficient; PSR, People Separation Reliability Coefficient.

The reliability of the hits and logits of the examinees was adequate, both in the total sample and in the subsamples of males and females; the Cronbach’s alpha and PRI coefficients clearly exceed the value of 0.70, which is an indicator of minimally acceptable reliability. However, reliability did not reach this rating in all subsamples corresponding to the school year; it was inadequate in year 3. In the case of the total sample, the strata index of the people (S_p_ = 2.94) indicated that three ranges of people with different levels in the language variable are reliably identifiable.

It should be noted that the percentage of people with a severe misfit (infit and/or outfit > 2) was moderately low (14.4%).

### Analysis of the Memory Variable With the Dichotomous Model

**Figure 2:**
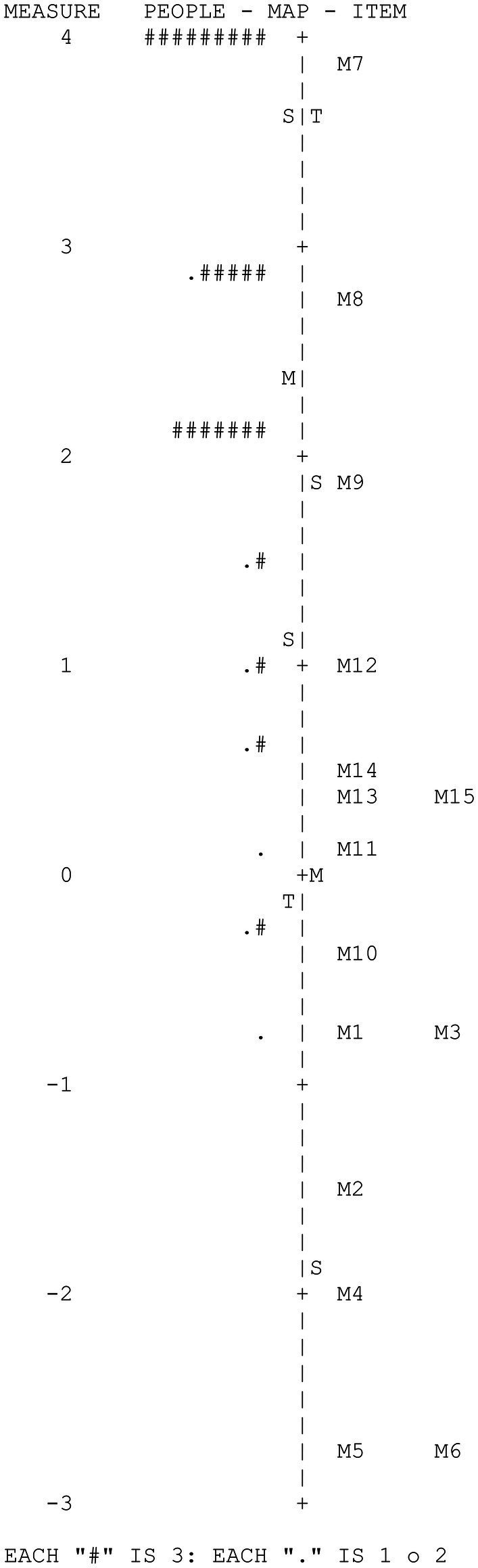
CAPALIST-memory. Variable map.

Figure 2 shows the Wright map corresponding to the memory variable. The map provides two basic results: (1) moderate variability of the participants and great variability of the items in the measured variable and (2) the low adaptation of the difficulty of some items to the levels of the examinees due to the extreme ease of such items.

Table 4 shows the values of the items. Despite the fact that the people sample size was not large, the difficulty parameters of the items were estimated with high precision; the reliability index of the items was high (IRI = 0.91). The strata index of the items (*S_i_* = 4.57) indicated that more than four ranges of items with different difficulties are reliably identifiable.

**Table 4:** CAPALIST-memory. Item properties.

Item	X	D	SE	Infit	Outfit	RiX
1.M1. Can you tell me your full name?	77	−0.72	0.46	0.84	0.36	0.44
2.M2. How old are you?	80	−1.55	0.62	0.95	0.34	0.30
3.M3. What is your mam’s/dad’s name?	77	−0.72	0.46	0.91	0.67	0.37
4.M4. Have you got any brothers or sisters? What are their names?//If he/she has no siblings, ask about a friend	81	−2.01	0.74	0.86	0.18	0.30
5.M5. What is your teacher’s name?	82	−2.75	1.02	1.06	0.99	0.08
6.M6. How do you get to school every day? (If he/she does not give an answer, give him/her options such as walking, by bus, by car…)	82	−2.75	1.02	0.98	0.26	0.18
7.M7. The last time you were given out to, what were you doing?	27	3.84	0.30	1.30	3.43	0.34
8.M8. Which is the last song that you have learnt? (ask the teachers about the last issues they have shown the children). Encourage free storytelling	41	2.76	0.27	0.99	0.99	0.57
9.M9. What did you do in class yesterday?	53	1.90	0.27	0.97	1.05	0.55
10.M10. Where was the bird?	75	−0.34	0.41	1.13	1.07	0.30
11.M11. How many girls where there?	72	0.11	0.37	0.86	0.69	0.48
12.M12. Do you remember the names of the children?	64	1.00	0.31	1.00	0.85	0.50
13.M13. What did the tree have?	70	0.37	0.35	0.84	0.64	0.52
14.M14. What were the children playing?	69	0.48	0.34	0.97	1.09	0.45
15.M15. Who had the ball?	70	0.37	0.35	0.98	0.79	0.46
Mean	68.0	0.00	0.48	0.98	0.89	0.44
SD	15.5	1.83	0.24	0.12	0.74	0.14

The difficulty of the items in logit varied between 3.84 and −2.75 (*M* = 0.00; *SD* = 1.83). Only item M7 presented an outfit value greater than 2, indicating a severe misfit with the model.

The principal components analysis of the residuals indicated that the subscale can be considered one dimensional, given that the measurements accounted for a sufficient percentage of the total variance (42.5%) and that the eigenvalue of the first component of the residuals were less than 3 (2.14). On the other hand, it is observed that only five of the 105 values of *Q*_3_ were higher than 0.20 (4.8%), which indicates that local dependence did not seriously affect the items. The maximum value of *Q*_3_ was 0.60 (correlation between the residuals of items M1 and M5). Furthermore, no gender-related DIF was detected in any of the items.

Table 5 shows the descriptive statistics of the scores of those evaluated; the scores ranged between 15 and 6 hits and 5.45 and −0.69 logits. The standard deviations of the hits and the logits were moderately high. It should be noted that the mean in logits of the participants (2.69) was much higher than the mean difficulty of the items (0.00), indicating that the test is very easy for the children evaluated. In fact, six extremely easy items were observed that were correctly solved by more than 90% of the evaluated participants (M1, M2, M3, M4, M5, and M6). Table 5 also shows the means on the logit scale of the groups formed according to gender and grade. The mean levels of females and males did not differ significantly, *t* (80) = 0.47, *p* = 0.64. However, performance grew significantly with the school grade, *F* (2, 80) = 14.82, *p* < 0.001. The mean of second-year students was higher than that of first-year students by 1.14 logits; this difference being significant, *t* (40) = 2.86, *p* < 0.01, and large effect size (*d* = 0.82); the mean of third-year students was higher than that of second-year students by 0.88 logits, this difference being significant, *t* (58) = 2.74 *p* < 0.01 and of medium effect size (*d* = 0.71).

**Table 5:** Memory. Descriptive statistics of the participants’ scores.

Group	Mean X	SD X	Max. X	Min. X	Alpha	Mean L	SD L	Max. L	Min. L	PSR
Total	12.3	2.1	15	6	0.66	2.69	1.53	5.45	−0.69	0.45
Male	12.2	2.2	15	6	0.68	2.62	1.59	5.45	−0.69	0.49
Female	12.4	2.0	15	7	0.65	2.78	1.45	5.45	−0.27	0.40
1st Year	10.5	2.5	15	6	0.65	1.53	1.49	5.45	−0.69	0.59
2nd Year	12.4	1.7	15	7	0.50	2.66	1.24	5.45	−0.27	0.28
3rd Year	13.5	1.1	15	12	0.25	3.54	1.22	5.45	2.13	0.00

X, hits; L, logit; Alpha, Cronbach’s alpha coefficient; PSR, People Separation Reliability Coefficient.

The reliability of the hits and logits of the examinees was low both in the total sample and in the subsamples formed according to gender and school grade: Cronbach’s alpha coefficient and PRI were clearly lower than the value of 0.70, indicative of minimally acceptable reliability. In the case of the total sample, the person strata index (*S_p_* = 1.54) indicated that at least two ranges of people with different levels in the memory variable could not be reliably identified. In other words, the poor adaptation of the difficulty of the items to the level of the people did not allow to differentiate accurately the performance in memory between them.

It should be noted that the percentage of people with a severe misfit (infit and/or outfit > 2) was moderately low (10.8%).

### Analysis of the Social Thinking Variable With the Dichotomous Model

**Figure 3:**
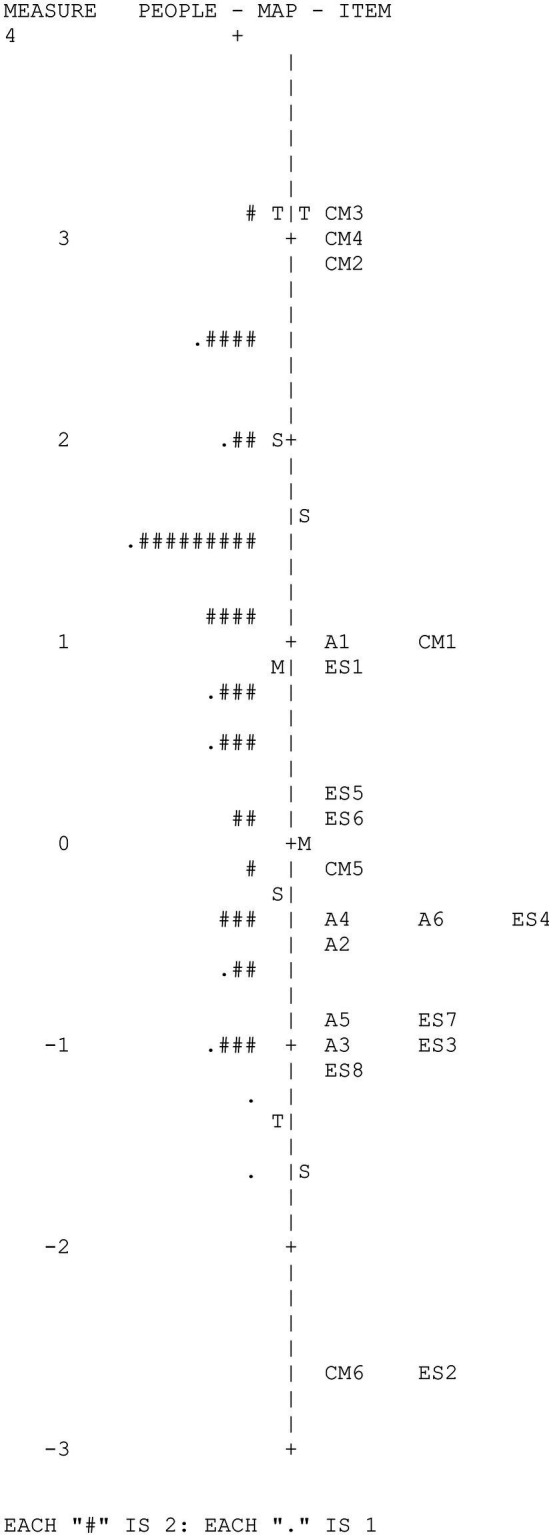
CAPALIST-social thinking. Variable map.

Figure 3 shows the Wright map corresponding to the social thinking variable. The map provides two basic results: (1) high variability of the participants and of the items in the measured variable and (2) the low adaptation of the difficulty of some items to the level of the examinees due to the extreme ease of such items.

Table 6 shows the values of the items. Although the sample size of the participants was not large, the difficulty parameters of the items were estimated with high precision; the reliability index of the items was high (IRI = 0.96). The strata index of the items (*S_i_* = 6.61) indicated that more than six ranges of items with different difficulties were reliably identifiable.

**Table 6:** CAPALIST- social thinking. Item properties.

Item	X	D	SE	Infit	Outfit	RiX
1. A1. Hey, (call him/her by another name)	40	0.96	0.25	1.15	1.22	0.34
2.A2. Hey, your teacher’s name is_____(give him/her a wrong name)	62	−0.51	0.28	0.98	1.25	0.41
3.A3. Hey, I can see a dog…. Do you know where it is? (while waiting for the child’s answer, interviewer stares at picture.) If the child finally does not give in to the suggestion, congratulate him/her and tell him/her you have made a mistake.	68	−1.04	0.31	1.07	1.17	0.32
4.A4.Color with the red color (and the interviewer hands him/her a different color and waits for the child to correct him/her or to pick up the red color). Say you are sorry for making a mistake	61	−0.44	0.28	0.78	0.61	0.62
5.A5. And the dog, where was the dog?	66	−0.85	0.30	1.00	0.96	0.41
6.A6. Where was the mouse in the picture?	61	−0.44	0.28	1.39	1.67	0.12
7.ES1. What do you think is going on in the picture? If he/she does not understand the question, point at the frightened man and ask: What do you think is wrong with him?	41	0.90	0.25	1.11	1.19	0.37
8.ES2. If he/she still does not understand the question….do you think the man is sad, angry or happy?	79	−2.69	0.53	1.04	1.16	0.18
9.ES3. Ask about the different emotions of the characters.	68	−1.04	0.31	1.27	1.59	0.14
10.ES4. Do you remember the last time you hurt yourself? What happened? How did you feel?	60	−0.36	0.28	0.73	0.58	0.66
11.ES5. If he/she answered the previous question….ask about: how do you think mam/dad/you brother/ your sister felt?	51	0.27	0.26	0.78	0.71	0.64
12.ES6. Do you remember the last time one of your classmates was punished in class?	54	0.07	0.26	0.94	1.00	0.50
13.ES7. (If he/she answers positively ES6): How do you think your friend felt?	66	−0.85	0.30	0.95	0.95	0.43
14.ES8. How do you think your teacher felt?	69	−1.14	0.32	0.90	0.73	0.46
CM1. Who has told the truth out of the three children?, remind the child that he/she was there and that nothing will happen to him/her but that he/she has to tell the truth because he/she is the only witness who has seen it all.	40	0.96	0.25	1.00	0.98	0.47
CM2. Who has lied?	14	2.83	0.31	0.96	0.76	0.39
CM3. Who do you think is going to get given out to by the man?	11	3.15	0.34	1.00	1.54	0.29
CM4. Why do you think “X” is going to get given out to?	12	3.03	0.33	0.94	0.67	0.39
CM5. Who really broke the window? What is the truth?	57	−0.14	0.27	0.98	0.92	0.48
CM6. And is that right or wrong?	79	−2.69	0.53	0.87	0.30	0.37
Mean	53.0	0.00	0.31	0.99	1.00	0.40
SD	20.2	1.58	0.08	0.15	0.35	0.15

The difficulty of the items in logit varied between 3.15 and −2.69 (*M* = 0.00; *SD* = 1.58). None of the items presented a severe misfit with the model.

The principal components analysis of the residuals indicated that the subscale can be considered one dimensional, given that the measurements accounted for a sufficient percentage of the total variance (39.9%) and that the eigenvalue of the first component of the residuals were less than 3 (2.29). On the other hand, it is observed that only 5 of the 190 values of *Q*_3_ were higher than 0.20 (2.6%), which indicates that local dependence did not seriously affect the items. The maximum value of *Q*_3_ was 0.39 (correlation between the residuals of items CM3 and CM4). Furthermore, no gender-related DIF was detected in any of the items.

Table 7 shows the descriptive statistics of the evaluated scores; the scores ranged between 18 and 5 hits and 3.15 and −1.57 logits. The standard deviations of the hits and the logits were moderately high. The mean in logits of the participants (0.85) moderately exceeded the mean difficulty of the items (0.00), indicating that the test was somewhat easy for the children evaluated. In fact, two extremely easy items are observed that were correctly solved by more than 90% of the evaluated individuals (ES2 and CM6). Table 7 also shows the means on the logit scale of the groups formed according to gender and grade. The mean levels of females and males did not differ significantly, *t* (77) = 0.57, *p* = 0.57. However, performance grew significantly with a school grade, *F* (2, 80) = 12.31, *p* < 0.001. The mean of second-year students was higher than that of first-year students by 0.59 logits, this difference being significant, *t* (49) = 2.01, *p* < 0.05, and of medium effect size (*d* = 0.57). The mean of third-year students was higher than that of second-year students by 0.79 logits, this difference being significant, *t* (53) = 2.94, *p* < 0.01, and of average effect size (*d* = 0.76).

**Table 7:** Social thinking. Descriptive statistics of the participants’ scores.

Group	Mean X	SD X	Max. X	Min. X	Alpha	Mean L	SD L	Max. L	Min. L	PSR
Total	12.8	3.4	18	5	0.73	0.85	1.14	3.15	−1.57	0.65
Male	12.6	3.4	18	5	0.75	0.79	1.12	3.15	−1.57	0.65
Female	12.9	3.3	18	7	0.73	0.93	1.16	3.15	−0.94	0.65
1st Year	10.6	3.1	15	6	0.63	0.12	0.92	1.52	−1.25	0.58
2nd Year	12.3	3.5	17	5	0.75	0.72	1.17	2.49	−1.57	0.67
3rd Year	14.7	2.3	18	8	0.51	1.50	0.86	3.15	−0.66	0.30

X, hits; L, logit; Alpha, Cronbach’s alpha coefficient; PSR, People Separation Reliability Index.

The reliability of the logit scores of the participants did not reach the minimally acceptable level both in the total sample and in the subsamples formed according to gender and grade; the PRI indices were slightly lower than the value of 0.70. However, Cronbach’s alpha coefficients slightly exceeded that level in some samples. It is known that the alpha coefficient tends to adopt values higher than PRI because it is calculated from scores that are not linear representations of the latent variable. Therefore, PRI is a more appropriate indicator of the reliability of the measures (Anselmini et al., 2019). In the case of the total sample, the people strata index (*S_p_* = 2.16) indicated that at least two ranges of people with different levels of the social thinking variable could be reliably identified.

Finally, it is observed that the percentage of people with a severe misfit (infit and/or outfit > 2) was low (4.8%).

### Analysis of the Orientation Variable With the Dichotomous Model

**Figure 4:**
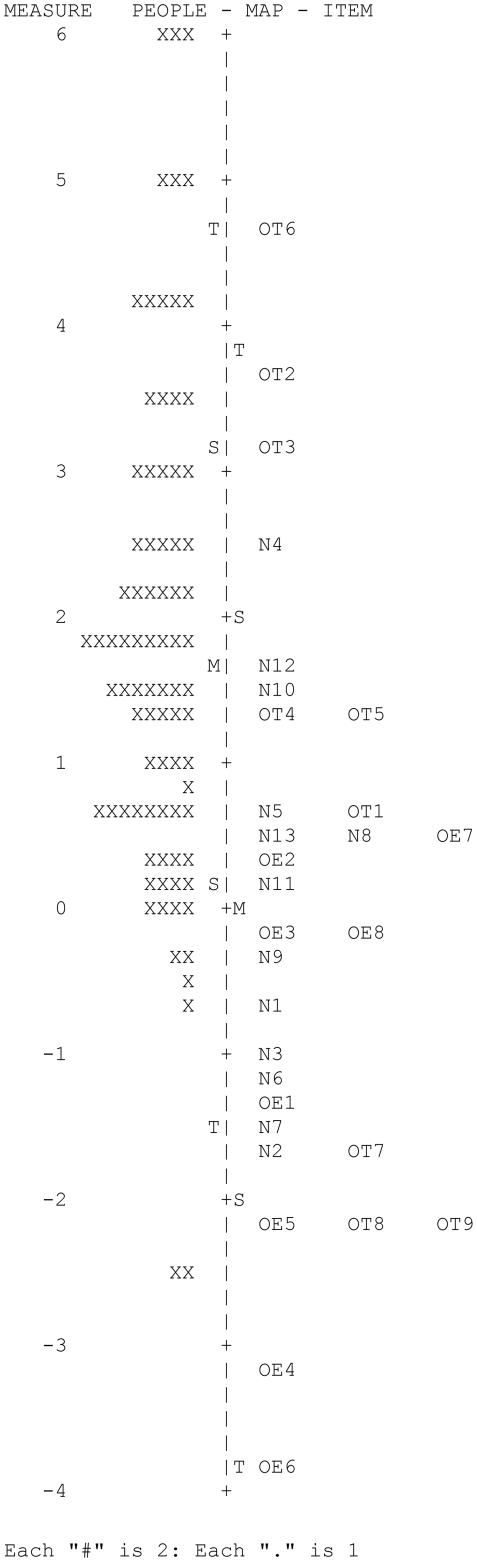
CAPALIST-contextual information. Variable map.

Figure 4 shows the Wright map corresponding to the orientation variable. The map provides two basic results: (1) great variability of the participants and of the items in the measured variable and (2) the low adaptation of the difficulty of some items to the level of the participants due to the extreme ease of such items.

Table 8 shows the values of the items. Although the participants’ sample size was not large, the difficulty parameters of the items were estimated with high precision; the reliability coefficient of the items was high (IRI = 0.95). The strata index of the items (*S_i_* = 6.04) indicated that six ranges of items with different difficulties were reliably identifiable.

**Table 8:** CAPALIST-contextual information. Item properties.

Item	X	D	SE	Infit	Outfit	RiX
1.OE1. Where are we now?	75	−1.31	0.42	1.03	3.23	0.23
2.OE2. Where is the nearest bathroom?	61	0.26	0.29	1.32	2.32	0.26
3.OE3. Where is your house?	65	−0.09	0.31	1.11	2.38	0.33
4.OE4. Can you see the bird? Is it over or under the house? Color it with whatever color you want.	81	−3.09	0.77	1.27	0.60	0.16
5.OE5. And the children, are they inside or outside the house?	79	−2.23	0.57	0.98	0.42	0.32
6.OE6. The ball, is it ton top of or under the foot?	82	−3.88	1.05	1.12	0.31	0.16
7.OE7. Which child is further away from the tree?	58	0.50	0.28	0.89	0.80	0.52
8.OE8. Which child is closest to the tree?	66	−0.18	0.31	1.12	1.02	0.37
9.OT1. Is it morning, afternoon or evening?	55	0.73	0.27	1.10	2.08	0.41
10.OT2. What is today’s date?	18	3.64	0.33	0.75	0.75	0.65
11.OT3. And what day of the week is it?	22	3.22	0.31	0.75	0.68	0.67
12.OT4. Have you had your breakfast/lunch/dinner yet?	47	1.31	0.26	1.26	1.30	0.40
13.OT5. ¿Sabes en qué estación del año estamos?	46	1.38	0.26	0.79	0.69	0.62
14.OT6. And what year is it?	11	4.61	0.42	0.71	0.48	0.65
15.OT7. In the picture, is it daytime or night time?	77	−1.70	0.47	0.92	1.14	0.31
16.OT8. If he/she is unsure…. When children play ball, is it day or night?	79	−2.23	0.57	0.66	0.25	0.41
17.OT9. Then, what will we draw, a sun or a moon? If you want, you can draw it	79	−0.2.23	0.57	0.66	0.25	0.41
18.N1. How many apples(X) are there? Count with the child (He/she can count up to….)	71	−0.72	0.35	0.89	0.61	0.45
19.N2. Are there a lot of or a few “apples, plums….”?	77	−1.70	0.47	0.94	1.12	0.30
20.N3. Where are there more “apples”? (here or here, left or right)	73	−0.99	0.38	0.86	0.48	0.45
21.N4. How many times do you eat each day?	30	2.53	0.28	1.21	1.31	0.46
22.N5. How many boys and girls did we say there was in the picture?	56	0.66	0.27	1.13	0.96	0.44
23.N6. With a few coloring pens outside the pencil case, and the rest inside it, ask: Where are there more colors, inside the pencil case or outside on the table?	74	−1.14	0.40	0.95	0.66	0.39
24.N7. Who is the oldest child in the picture?	76	−1.49	0.44	1.04	0.74	0.34
25.N8. Who is the youngest child in the picture?	57	0.58	0.28	0.81	0.63	0.58
26.N9. What do you do first, eat or brush your teeth?	68	−0.38	0.32	1.13	1.24	0.34
27.N10. What clothes do you put on first? (if he/she does not answer, give him/her options in the wrong order)	44	1.52	0.26	1.12	1.55	0.45
28.N11. To drink water, what do you have to do?	62	0.18	0.29	0.96	0.79	0.48
29.N12. If I had to wash my teeth, what would I have to do? (step by step)	42	1.65	0.26	0.85	0.76	0.61
30.N13. What do you do before you go to bed to sleep?	57	0.58	0.28	0.85	0.71	0.55
Mean	59.6	0.00	0.39	0.97	1.01	0.42
SD	19.3	1.95	0.17	0.18	0.68	0.14

The difficulty of the items in logit varied between 4.61 and −3.88 (*M* = 0.00; *SD* = 1.95). Four items presented a severe misfit with the model (OE1, OE2, OE3, and OT1).

The principal components analysis of the residuals indicated that the subscale can be considered one dimensional, since the measurements accounted for a sufficient percentage of the total variance (46.7%) and that the eigenvalue of the first component of the residuals were less than 3 (2.60). On the other hand, it is observed that only 11 of the 435 values of *Q*_3_ were higher than 0.20 (2.5%), which indicated that local dependence did not seriously affect the items. The maximum value of *Q*_3_ was 0.47 (correlation between the residuals of items OE6 and OT8). Furthermore, no gender-related DIF was detected in any of the items.

Table 9 shows the descriptive statistics of the scores of the participants; the scores ranged between 30 and 5 hits and 6.46 and −2.53 logits. The standard deviations of the hits and the logits were high. The mean in logits of the participants (1.79) clearly exceeded the average difficulty of the items (0.00), indicating that the test was very easy for the children evaluated. In fact, eight extremely easy items were observed that were correctly solved by more than 90% of the participants evaluated (OE4, OE5, OE6, OT7, OT8, OT9, N2, and N7). Table 9 also shows the means on the logit scale of the groups formed according to gender and grade. The average levels of females and males did not differ significantly, *t* (80) = 1.02, *p* = 0.31. However, performance grew significantly with the school grade, *F* (2, 80) = 26.22, *p* < 0.001. The mean of second-year students was higher than that of first-year students by 1.14 logits, this difference being significant, *t* (45) = 3.44, *p* < 0.001, and with a large effect size (*d* = 0.99). The mean of third-year students was higher than that of second-year students by 1.59 logits, this difference being significant, *t* (54) = 4.32, *p* < 0.001, and with a large effect size (*d* = 1.12).

**Table 9:** Contextual information. Descriptive statistics of the participants’ scores.

Group	Mean X	SD X	Max. X	Min. X	Alpha	Mean L	SD L	Max. L	Min. L	PSR
Total	21.5	5.2	30	5	0.86	1.79	1.74	6.46	−2.53	0.82
Male	20.9	5.7	30	5	0.88	1.61	1.83	6.46	−2.53	0.85
Female	22.3	4.4	30	12	0.82	2.00	1.61	6.46	−0.72	0.78
1st Year	16.7	4.2	28	5	0.75	0.36	1.14	4.11	−2.53	0.79
2nd Year	21.3	4.3	28	5	0.81	1.50	1.17	4.11	−2.53	0.75
3rd Year	25.3	3.4	30	17	0.76	3.09	1.63	6.46	0.37	0.66

X, hits; L, logit; Alpha, Cronbach’s alpha coefficient; PSR, People Separation Reliability Index.

The reliability of the hits and logit scores of the participants was adequate both in the total sample and in most of the subsamples formed according to gender and grade (although in the third-year subsample, PRI was slightly lower than 0.70). In the case of the total sample, the strata index of the people (*S_p_* = 3.2) indicated that at least three ranges of people with different levels in the variable orientation could be reliably identified.

It should be noted that the percentage of people with a severe misfit (infit and/or outfit > 2) was low (8.4%).

## Discussion

Using two invariant measurement models, the four subscales of the CAPALIST protocol (assessment of testifying capabilities) were analyzed. Given the polytomous structure of the original response categories, the rating scale model (Andrich, 1978) was used initially in order to determine the performance of these categories. The results indicate that in three of the four subscales (memory, social thinking, and contextual information), the thresholds between the successive categories were not ordered monotonically, indicating that the central category is not modal (it is not the most probable in any range of the measured variable). Therefore, a dichotomous recoding was performed by grouping the two lower categories. In order for the new numerical scale to be homogeneous in the four subscales, the values were recoded as follows: 1 = masters the ability (old category 3); 0 = does not adequately master the ability (old categories 1 and 2). The data obtained with the recoding were analyzed with the Rasch dichotomous model.

Taken together, the four variables did not severely violate the requirements of the model. The principal component analysis of the residuals indicated that the scales are fundamentally one-dimensional and that, given the scarce presence of dependent items, the assumption of local independence was not severely violated. Likewise, the presence of differential performance associated with the gender of the participants was not detected. In addition, the percentage of people who presented responses that were mismatched with the model (infit and/or outfit > 2) was low; the values ranged between 14.4% (language) and 4.8% (social thinking). The number of items that were severely mismatched (infit and/or outfit > 2) was also low: language (L10 and L11), memory (M7), and contextual orientation (OE1, OE2, OE3, and OT1). It is recommended to replace these items in a future version of CAPALIST.

Despite the fact that the sample size of participants was not high, the difficulty parameters of the items were estimated with good precision. The item reliability indices (IRI) were high: language (0.94), memory (0.91), social thinking (0.96), and contextual information (0.95). The number of strata of different difficulties (*S_i_*) indicated that the continuum of difficulty has been adequately sampled: language (5.54), memory (4.57), social thinking (6.61), and contextual information (6.04).

The impact on gender and school grade scales was as expected from the theoretical point of view; no significant differences appeared between the means of males and females on any of the scales. In addition, a significant increase in means was observed in successive courses, with the effect size being medium or large in the increments.

The strengths of the test so far: The poor reliability of the participants’ scores on some subscales is the main weakness. To assess the precision, the specific statistics of the Rasch model were used. Cronbach’s alpha coefficient, usually used in classical analyses, tends to adopt values higher than PRI as it is calculated from scores that are not linear representations of the latent variable. Therefore, the person reliability index (PRI) is considered a more appropriate indicator of the reliability of the measures (Anselmini et al., 2019). From PRI, other statistics are defined, such as the separability index (*G*) and the strata index (*S*). The latter indicates the number of categories or ranges of people with different levels in the variable that the test allows to identify (Bond and Fox, 2015). The results reveal that the reliability of two of the subscales is adequate: language (PRI = 0.79; *S_p_* = 2.94) and contextual information (PRI = 0.82; *S_p_* = 3.2). However, in the memory subscale, reliability is low (PRI = 0.45; *S_p_* = 1.54), while in the social thinking subscale, reliability is moderately low (PRI = 0.65; *S_p_* = 2.16).

The low reliability of the scales is mainly due to their excessive ease and the low number of items with a difficulty appropriate to the level of the individuals analyzed. In the memory subscale, the people’s mean is much higher than 0 (the mean difficulty of the items). Furthermore, if very easy items are excluded (solved correctly in more than 90% of the cases), the number of “functional” items in this scale is low (9).

Consequently, to increase reliability, it is advisable to substitute very easy items for others of greater difficulty and to increase the number of items on the shorter scales.

There are two fundamental limitations in the present study: the size of the study sample and the absence of a data collection design with several interviewers. The responses to the CAPALIST items are scored by interviewers or raters whose performance is a known source of error that must be quantified (Eckes, 2009). Both limitations must be mitigated in future studies.

## Conclusion

The present study has demonstrated an acceptable performance of CAPALIST to assess relevant testifying abilities (language, memory, contextual information, and social thinking), although the results can help in decision-making for optimization of the set of items presented. In terms of police procedures, this is not an easy decision. Some questions, although answered correctly by the majority of participants, provide valuable information that is difficult not to contrast in all cases. CAPALIST can assist in the specific assessment of relevant capabilities to testify in those cases where they are suspected of being affected. In this way, it will be possible to adapt the procedures for obtaining statements to the skills of the witnesses. The presence or absence of credibility criteria can also be evaluated according to their cognitive characteristics and not based on population stereotypes. For example, to the extent that a child witness between the ages of 3 and 6 has problems managing the time dimension or quantifying how many times an event has occurred, questions about these aspects should be avoided since the information provided may not be valid. If the child has provided information on these elements, when assessing inconsistencies with police evidence, we must take into account his or her ability. That is, the incongruity with other evidence could not be due to the fact that the events did not take place but rather to the cognitive characteristics of the possible victim.

The future challenge should focus on the application of the instrument with other samples in great need of this type of procedure, such as with victims with intellectual disabilities (Silva et al., 2016). In participants with some type of intellectual disability, it would be necessary to verify whether the memory items can be ignored, as they have shown an improbable fit because they are easily solved. We recommend caution when applying it to samples not evaluated by this study but also recommend taking advantage of future studies to propose the analysis of new questions, especially those related to the memory variable.

## Data Availability Statement

The raw data supporting the conclusions of this article will be made available by the authors, without undue reservation.

## Ethics Statement

The studies involving human participants were reviewed and approved by Comité de ética de la Universidad Complutense de Madrid. Written informed consent to participate in this study was provided by the participants’ legal guardian/next of kin.

## Author Contributions

MC, GP, ES, JG, and AM: conceptualization, investigation and writing, reviewing, and editing. MC, GP, ES, and AM: methodology. ES: project administration. GP and ES: formal analysis and writing the original draft. MC and AM: supervision. GP: data curation. MC: funding acquisition. All authors contributed to the article and approved the submitted version.

### Conflict of Interest

The authors declare that the research was conducted in the absence of any commercial or financial relationships that could be construed as a potential conflict of interest.

## Footnotes

^1^The procedure can be seen in https://www.researchgate.net/publication/344782111_Short_Procedure_for_the_Assessment_of_the_Abilities_to_Testify

## Supplementary Material

Data Sheet 1 - Appendix I

Data Sheet 2 - Appendix II
