# Supplementary material for: Assessment of Testifying Ability in Preschool Children: CAPALIST
Source: Front Psychol. 2021 Jul 16;12:662630. doi: 10.3389/fpsyg.2021.662630 (PMC8322118; doi:10.3389/fpsyg.2021.662630)
Supplement: Supplementary file 1 [file Data_Sheet_1.pdf]

APPENDIX I. *CAPALIST*

INITIAL PRESENTATION		MALE / FEMALE:	
Hello, (as you already know) my name is …… and his/her name is …. Today, we are going to draw/color this drawing with you, do you like it? (The drawing and the colored pencils are placed on the table… the child is left to organize him/herself. <u>Assign the values of each category</u>			
MEMORY			
Questions	Category	0<br>Does not have the ability	1<br>Masters the ability
M1. Can you tell me your full name?	MEMORY		
M2. How old are you?	MEMORY		
M3. What is your mam's / dad's name?	MEMORY		
M4. Have you got any brothers or sisters? What are their names? // If he/she has no siblings, ask about a friend	MEMORY		
M5. What is your teacher's name?	MEMORY		
M6. How do you get to school every day? (If he/she doesn't give an answer, give him/her options such as walking, by bus, by car…)	MEMORY		
M7. The last time you were given out to, what were you doing?	MEMORY		
SPATIAL AND TEMPORAL (CONTEXTUAL) INFORMATION			
Questions	Category	0<br>Does not have the ability	1<br>Masters the ability
OE1. Where are we now?	CONTEXT		
OE2. Where is the nearest bathroom?	CONTEXT		
OE3. Where is your house?	CONTEXT		
OT1. Is it morning, afternoon or evening?	CONTEXT		
OT2. What is today's date?	CONTEXT		
OT3. And what day of the week is it?	CONTEXT		
OT4. Have you had your breakfast/lunch/dinner yet?	CONTEXT		
OT5. ¿Sabes en qué estación del año estamos?	CONTEXT		
OT6. And what year is it?	CONTEXT		
A1. Hey, (*call him/her by another name*)………	SOCIAL THINKING		
LANGUAGE			

Questions	Category	0	1
		Does not have the ability	Masters the ability
L1. What is your teacher like? (Minimum 2 elements)	LANGUAGE		
L2. Now, close your eyes and describe me (description of the interviewer Minimum 2 elements)	LANGUAGE		
L3. Can you tell me what that girl is wearing? Start from the bottom. (If he/she doesn't know how, interviewer will point items out)	LANGUAGE		
L4. Tell me which ones are boys and which are girls in this picture (give them names)	LANGUAGE		
L5. Who is the tallest?	LANGUAGE		
L6. And the shortest?	LANGUAGE		
L7. Coloring the picture…. What is this color? And this one? (minimum 5 colors)	LANGUAGE		
L8. What can you see in the picture? Minimum 5 elements	LANGUAGE		
A2. Hey, your teacher's name is_____(give him/her a wrong name)	SOCIAL THINKING		
L9. And this boy, can you tell me what this boy is wearing? Start from the bottom. (If he/she doesn't know how, interviewer will point items out). Minimum X elements	LANGUAGE		
L10. Now I'm going to point out parts of the children's bodies and you must tell me what they are called, ok?	LANGUAGE		
L11. Where are the children in the picture?	LANGUAGE		
L12. And in this picture, can you describe what the house is like?	LANGUAGE		
A3. Hey, I can see a dog…. Do you know where it is? (while waiting for the child's answer, interviewer stares at picture.) If the child finally does not give in to the suggestion, congratulate him/her and tell him/her you have made a mistake.	SOCIAL THINKING		
L13. What is that in the tree?	LANGUAGE		
L14. What things are there in your class?	LANGUAGE		
L15. What things are there in your bedroom?	LANGUAGE		
L16. Describe the gym at your school	LANGUAGE		
L17. What is the supermarket where you go shopping with mam and dad like?	LANGUAGE		
L18.What is the park that you go to play like?	LANGUAGE		
L19. What is in your backpack?	LANGUAGE		
L20. What is your jacket/coat/jumper like?	LANGUAGE		
L21. What is the bathroom that is closest to your classroom like?	LANGUAGE		
L22. Describe a kitchen	LANGUAGE		

NUMBER (QUANTITY AND ORDER)			
Questions	Category	0<br>Does not have the ability	1<br>Masters the ability
N1. How many apples(X) are there? Count with the child (He/she can count up to…..)	CONTEXT		
N2. Are there a lot of or a few “apples, plums….”?	CONTEXT		
N3. Where are there more “apples”? (here or here, left or right)	CONTEXT		
N4. How many times do you eat each day?	CONTEXT		
A4. Color with the red color (and the interviewer hands him/her a different color and waits for the child to correct him/her or to pick up the red color)<br>Say you are sorry for making a mistake	SOCIAL THINKING		
N5. How many boys and girls did we say there was in the picture?	CONTEXT		
N6. With a few coloring pens outside the pencil case, and the rest inside it, ask: Where are there more colors, inside the pencil case or outside on the table?	CONTEXT		
N7. Who is the oldest child in the picture?	CONTEXT		
N8. Who is the youngest child in the picture?	CONTEXT		
N9. What do you do first, eat or brush your teeth?	CONTEXT		
N10. What clothes do you put on first? (if he/she doesn’t answer, give him/her options in the wrong order)	CONTEXT		
N11. To drink water, what do you have to do?	CONTEXT		
N12. If I had to wash my teeth, what would I have to do? (step by step)	CONTEXT		
N13. What do you do before you go to bed to sleep?	CONTEXT		

ORIENTATION REASONING (Spatial Temporal)			
Questions	Category	0<br>Does not have the ability	1<br>Masters the ability
OT7. In the picture, is it daytime or night time?	CONTEXT		
OT8. If he/she is unsure…. When children play ball, is it day or night?	CONTEXT		
OT9. Then, what will we draw, a sun or a moon? If you want, you can draw it	CONTEXT		
OE4. Can you see the bird? Is it over or under the house? Color it with whatever color you want.	CONTEXT		

OE5. And the children, are they inside or outside the house?	CONTEXT		
OE6. The ball, is it ton top of or under the foot?	CONTEXT		
OE7. Which child is further away from the tree?	CONTEXT		
OE8. Which child is closest to the tree?	CONTEXT		
MEMORY 2			
Questions	Category	0. Does not have the ability	1. Masters the ability
M8. Which is the last song that you have learnt? (ask the teachers about the last issues they have shown the children). Encourage free storytelling	MEMORY		
M9. What did you do in class yesterday?	MEMORY		
The interviewer places the picture upside down and asks the child to recall what was in the picture, without looking at it. (Minimum: explain the main scene)	Category	0	1
		Does not have the ability	Masters the ability
M10. Where was the bird?	MEMORY		
M11. How many girls where there?	MEMORY		
M12. Do you remember the names of the children?	MEMORY		
M13. What did the tree have?	MEMORY		
M14. What were the children playing?	MEMORY		
M15. Who had the ball?	MEMORY		
A5. And the dog, where was the dog?	SOCIAL THINKING		
IDENTIFICATION SUBJECTIVE STATES			
Questions	Category	0	1
		Does not have the ability	Masters the ability
ES1. What do you think is going on in the picture? If he/she does not understand the question, point at the frightened man and ask: What do you think is wrong with him?	SOCIAL THINKING		
ES2. If he/she still does not understand the question….do you think the man is sad, angry or happy?	SOCIAL THINKING		
ES3. Ask about the different emotions of the characters.	SOCIAL THINKING		
ES4. Do you remember the last time you hurt yourself? What happened? How did you feel?	SOCIAL THINKING		

ES5. If he/she answered the previous question….ask about: how do you think mam/dad/you brother/ your sister felt?	SOCIAL THINKING		
ES6. Do you remember the last time one of your classmates was punished in class?	SOCIAL THINKING		
ES7. (If he/she answers positively ES6): How do you think your friend felt?	SOCIAL THINKING		
ES8. How do you think your teacher felt?	SOCIAL THINKING		
STORY OF THE PICTURE<br>The three children are playing ball and the oldest of the three (whatever name the child has given for him) kicks it very hard… so hard that the ball hits a window and breaks it. The man comes out of the house a little angry about the surprise and the fright, and as he sees the three children there and he has not seen who it was, he asks them one by one who has broken his window". Here, the child is reminded that he/she is behind the tree and that he/she has seen everything.<br>First he asks the oldest of the three (his name is used), and he says: It was not me, it was the youngest (the name is used). Then he asks the youngest (name) and he says: It was not me, it was the oldest (name). Finally, he asks the middle child (name) and he says: It was not me, it was the youngest (name).			
MORAL ABILITY (TRUTH/LIE GOOD/BAD)			
Questions	Category	0<br>Does not have the ability	1<br>Masters the ability
CM1. Who has told the truth out of the three children?, remind the child that he/she was there and that nothing will happen to him/her but that he/she has to tell the truth because he/she is the only witness who has seen it all.	SOCIAL THINKING		
CM2. Who has lied?	SOCIAL THINKING		
CM3. Who do you think is going to get given out to by the man?	SOCIAL THINKING		
CM4. Why do you think "X" is going to get given out to?	SOCIAL THINKING		
CM5. Who really broke the window? What is the truth?	SOCIAL THINKING		
CM6. And is that right or wrong?	SOCIAL THINKING		
Explain that the man is not going to punish any of them; he only wants them to be careful so that they don't hurt themselves.	SOCIAL THINKING		
A6. Where was the mouse in the picture?	SOCIAL THINKING		
