# Supplementary figures and images for: Assessment of Testifying Ability in Preschool Children: CAPALIST
Source: Front Psychol. 2021 Jul 16;12:662630. doi: 10.3389/fpsyg.2021.662630 (PMC8322118; doi:10.3389/fpsyg.2021.662630)

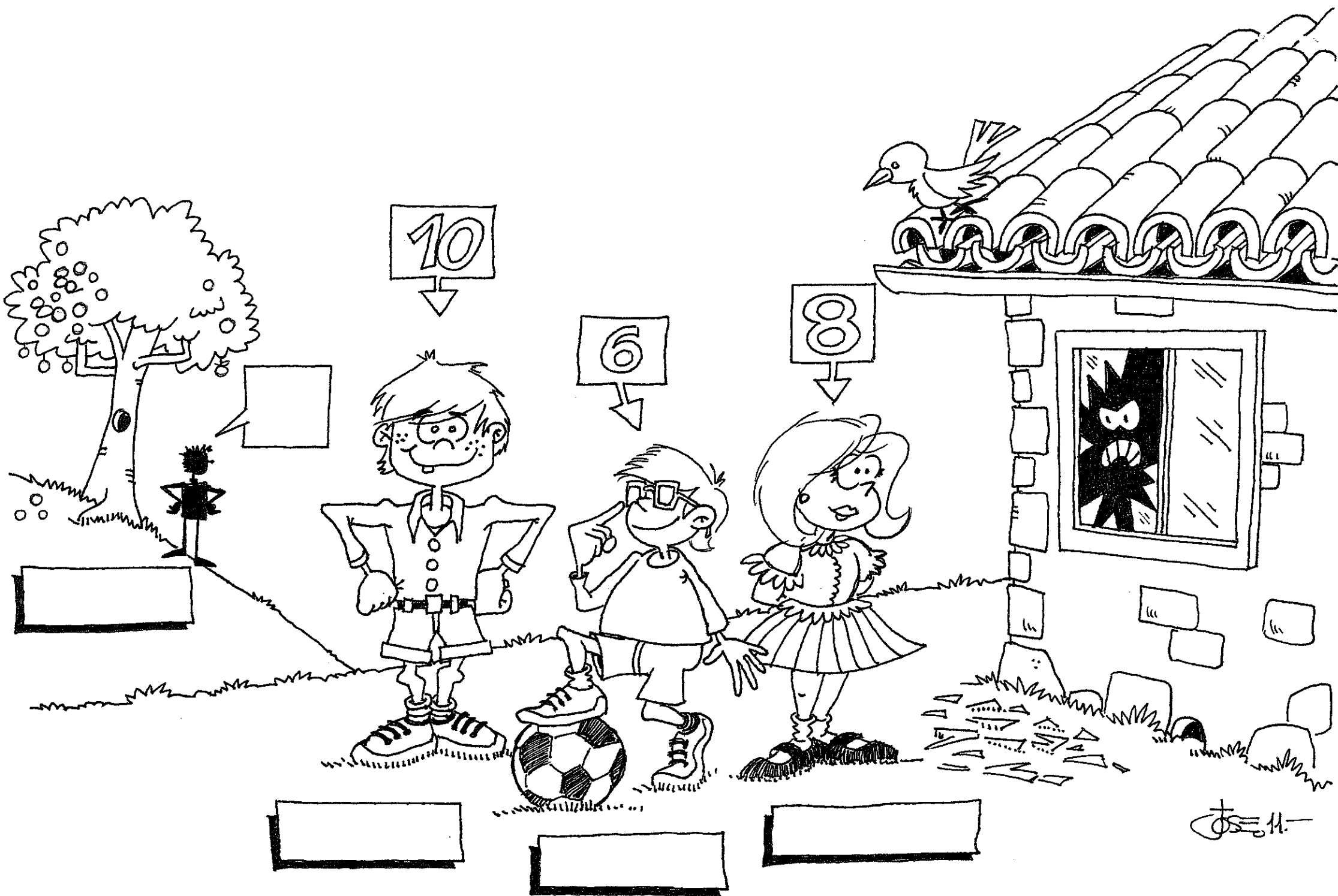

Supplement: Supplementary file 2 [file Data_Sheet_2.pdf]

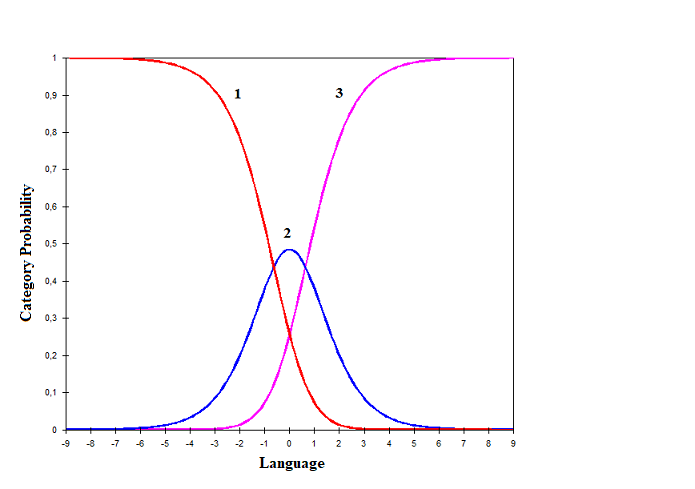

Supplement: Supplementary file 3 [file Image_1.PNG]

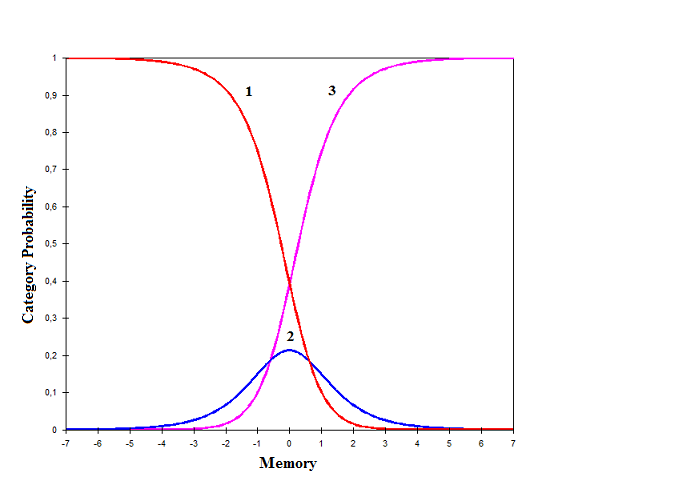

Supplement: Supplementary file 4 [file Image_2.PNG]

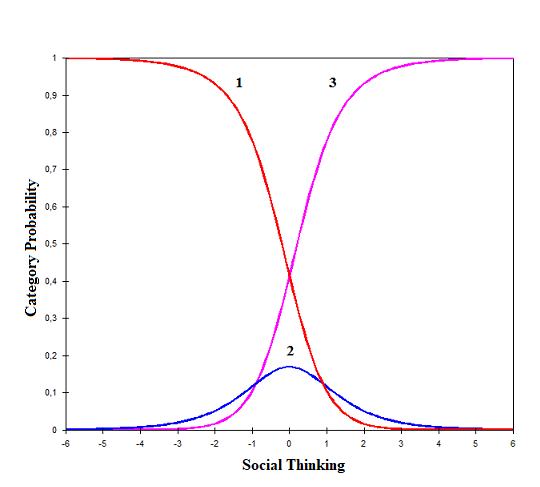

Supplement: Supplementary file 5 [file Image_3.PNG]

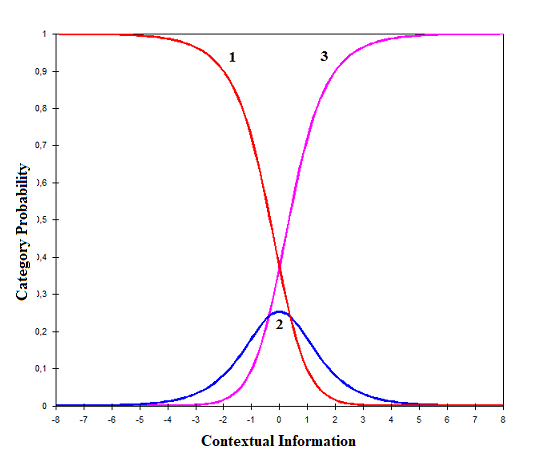

Supplement: Supplementary file 6 [file Image_4.PNG]
